# Changes in Transportation During the COVID-19 Pandemic: Results From a Survey of Middle-Aged and Older Floridians

**DOI:** 10.1093/geroni/igab046.488

**Published:** 2021-12-17

**Authors:** Anne Barrett, Cherish Michael, Jessica Noblitt

**Affiliations:** Florida State University, Tallahassee, Florida, United States

## Abstract

The pandemic’s numerous effects on everyday life include reductions in driving and changes in the use of other transportation modes, like getting rides from family and friends, walking, and biking. Aside from broad patterns, however, little is known about these changes, including how they affected different groups of the population and how they felt about them. Our study addresses these issues using data collected from an online survey of over 4,000 Floridians aged 50 or older, conducted between December 2020 and April 2021 and funded by the Florida Department of Transportation to support its aging road user program, Safe Mobility for Life. Changes in driving and in rides from family and friends were more striking than those in other transportation modes. Nearly 30 percent of respondents decreased their driving during the pandemic, while 20 percent got fewer rides from family and 25 percent got fewer rides from friends. In contrast, only 11 percent decreased their walking, and the same percentage increased it. Less common were changes in biking, with percent 7 decreasing and only 4 percent increasing it. Multivariate analyses revealed that these changes were influenced by gender, race, age, socioeconomic status, and health. Further insight was gained from analysis of an open-ended item, revealing positive and negative assessments of the changes. Positive assessments centered on feeling satisfied with working at home, spending more time outdoors, having more free time, and saving money. Negative assessments centered on social isolation, dissatisfaction with government responses to the pandemic, and reduced transportation options.

